# The thromboelastometric discrepancy between septic and trauma induced disseminated intravascular coagulation diagnosed by the scoring system from the Japanese association for acute medicine

**DOI:** 10.1097/MD.0000000000004514

**Published:** 2016-08-07

**Authors:** Hiroyuki Koami, Yuichiro Sakamoto, Ryota Sakurai, Miho Ohta, Hisashi Imahase, Mayuko Yahata, Mitsuru Umeka, Toru Miike, Futoshi Nagashima, Takashi Iwamura, Kosuke Chris Yamada, Satoshi Inoue

**Affiliations:** Advanced Emergency Care Center, Saga University Hospital, Saga city, Saga, Japan.

**Keywords:** disseminated intravascular coagulation, fibrinogen, sepsis, thromboelastometry, trauma

## Abstract

The aim of this study is to evaluate the hematological differences between septic and traumatic disseminated intravascular coagulation (DIC) using the rotational thromboelastometry (ROTEM).

This retrospective study includes all sepsis or severe trauma patients transported to our emergency department who underwent ROTEM from 2013 to 2014. All patients were divided into 2 groups based on the presence of DIC diagnosed by the Japanese Association for Acute Medicine (JAAM) DIC score. We statistically analyzed the demographics, clinical characteristics, laboratory data, ROTEM findings (EXTEM and FIBTEM), and outcome.

Fifty-seven patients (30 sepsis and 27 severe trauma) were included in primary analysis. Sepsis cases were significantly older and had higher systemic inflammatory response syndrome (SIRS) scores, whereas there were no significant differences in other parameters including Acute Physiology and Chronic Health Evaluation (APACHE) II score, sequential organ failure assessment (SOFA) score. Twenty-six patients (14 sepsis and 12 severe trauma) were diagnosed with DIC. The Septic DIC (S-DIC) group was significantly older and had higher DIC scores than the traumatic DIC (T-DIC) group. Hematologic examination revealed significantly higher CRP, fibrinogen, lower FDP, DD, and higher FDP/DD ratio were found in the S-DIC group in comparison with the T-DIC group. ROTEM findings showed that the A10, A20, and MCF in the FIBTEM test were significantly higher in the S-DIC group. However, no statistical differences were confirmed in the LI30, LI45, and ML in EXTEM test.

The plasma fibrinogen level and fibrinogen based clot firmness in whole-blood test revealed statistical significance between septic and traumatic DIC patients.

## 1 Introduction

Disseminated intravascular coagulation (DIC) is an unbalanced association between coagulation and fibrinolysis, which is encountered in cases of severe underlying illness.^[1]^ Sepsis and trauma are some of the most challenging therapeutic targets for emergency physicians and intensivists around the world. The fundamental approach to treat DIC is to control causative lesions, but mortality of DIC remains high at 31% to 48%, regardless of its cause.^[2–5]^

Guidelines for DIC published over the decades are shedding light on the efficacy of diagnosis and intervention.^[6–8]^ The newly established Japanese Association for Acute Medicine (JAAM) DIC score more accurately detected patients requiring definitive therapy for DIC, and was better able to predict the poor prognosis in patients with severe sepsis in comparison with other diagnostic criteria by the Japanese Ministry of Health and Welfare (JMHW) and the International Society of Thrombosis and Hemostasis (ISTH).^[4,8]^ It also is able to diagnose traumatic DIC effectively in the early phase with a higher sensitivity than the other criteria.^[5]^

Thus, we utilize this criterion to easily diagnose DIC in the emergency department (ED). However, it is often difficult to accurately grasp a patients’ underlying causes of DIC based only on DIC scores, such as a patient with both sepsis and trauma. The best example of this dilemma is severe sepsis (or septic DIC) cases with acute traumatic DIC. Bleeding complications delay the initiation of anticoagulation/inflammation therapy even if the patient's DIC score indicates septic DIC. Unfortunately, there is no global consensus or gold standard available for these situations.

Rotational thromboelastometry (ROTEM; TEM International, GmbH, Munich, Germany), is a point-of-care testing device using whole blood samples which has been increasingly used in various fields of medicine today.^[9–11]^ This device can immediately detect every phase of blood clotting and subsequent fibrinolysis. However, there are few studies investigating whether the ROTEM analysis enables us to understand DIC types derived from different underlying diseases.

The aim of this study is to evaluate the differences in the coagulation and fibrinolytic system between septic and traumatic DIC using ROTEM.

## 2 Materials and methods

### 2.1 Patients and laboratory sampling

This retrospective study has been approved by the Institutional Review Board (20140908&20150115). Saga University Hospital, which is a referral center in our region, has 7204 emergency department visits and 4278 transportations by ambulance car in 1 year. All sepsis or trauma patients transported to our hospital by an ambulance with ROTEM performed in the ED from January 2013 to December 2014 were enrolled in this study. The patients with out-of-hospital cardiac arrest, skin burn, and electrical injury were excluded. Length of hospital stay (LOS) of less than 2 days and milder trauma (injury severity score (ISS) of less than 16) were also excluded. Sepsis was defined as infection plus more than 2 parameters of systemic inflammatory response syndrome (SIRS) due to systemic infection at admission.^[12,13]^ All patients were screened for a diagnosis of DIC according to the JAAM DIC scoring system, which includes the presence of SIRS, abnormal value of platelets (Plt), international normalized ratio of prothrombin time (PT-INR), and fibrinogen and fibrin degradation products (FDP).^[8,14]^ The DIC was diagnosed when the total score was 4 or more (range: 0–8). We analyzed the demographics and clinical characteristics (age, sex, vital signs, SIRS score, JAAM DIC score, acute physiology and chronic health evaluation (APACHE) II score, sequential organ failure assessment (SOFA) score, laboratory data (white blood cell (WBC), hemoglobin (Hb), Plt, C-reactive protein (CRP), PT-INR, activated partial thromboplastin time (APTT), fibrinogen (Fib), FDP, D-dimer (DD), FDP to DD (FDP/DD) ratio, antithrombin III (ATIII), pH, base excess (BE), and lactate (Lac)), ROTEM findings and clinical outcomes (LOS and hospital mortality). All blood samples were collected upon admission. Site of infection in sepsis patients and various trauma scores (abbreviated injury scale (AIS), ISS, revised trauma score (RTS), and probability of survival (Ps)) were analyzed from the medical records, retrospectively.

### 2.2 ROTEM analysis

Our thromboelastometric evaluation was focused on the extrinsic coagulation pathway (EXTEM). The EXTEM test demonstrated that citrated whole blood was activated with tissue factor in a small disposable cuvette. We also used the FIBTEM test, which reflected the function of fibrinogen in the extrinsic pathway. Hyperfibrinolysis was diagnosed by improvement of fibrinolysis in the APTEM test, which used citrated whole blood with tissue factor and aprotinin. ROTEM parameters analyzed in this study included the clotting time (CT), the clot formation time (CFT), the alpha angle (α), the amplitude at 10 minutes (A10), 20 minutes after CT (A20), the maximum clot firmness (MCF), the lysis index at 30 minutes (LI30), 45 minutes (LI45), maximum lysis (ML), and the percentage of hyperfibrinolysis. All tests were continued for at least 60 minutes.

### 2.3 Statistical analysis

All continuous variables between each group are represented as median [quartile Q1, Q3] and categorical variables as percentages. The *P* values were evaluated from the Mann–Whitney *U* test for continuous variables and Fisher exact test, and *χ*^2^ tests were used for categorical variables. Values of *P* < 0.05 were considered to be significant. The data were statistically analyzed using the IBM SPSS for Windows version 22.0 (SPSS Inc, Chicago, IL).

## 3 Results

**Figure 1:**
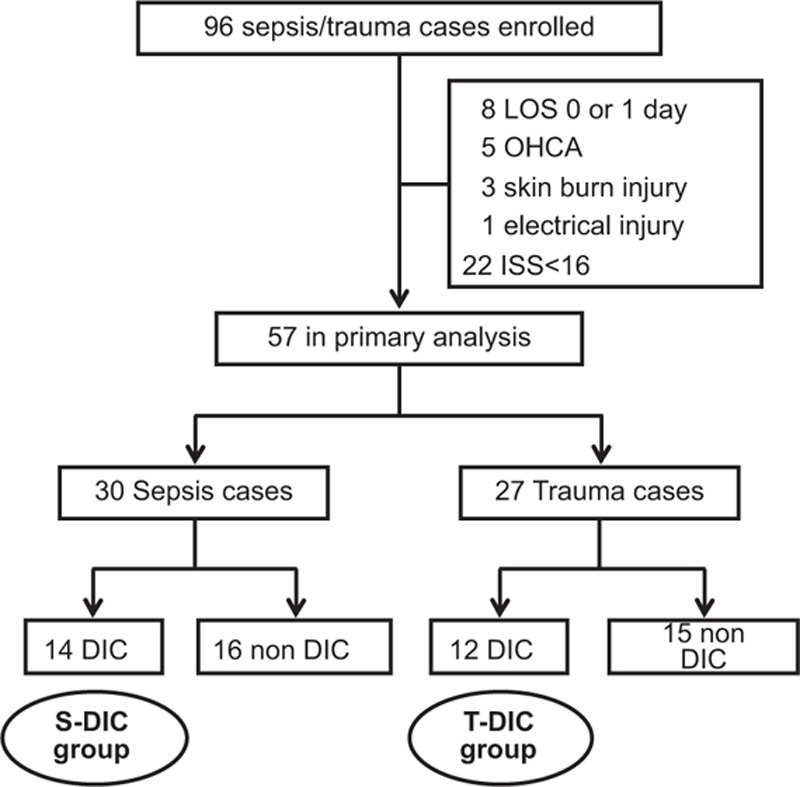
Study design. Ninety-six cases were matched with the inclusion criteria in this study, and 39 of them were excluded for different reasons. Primary analysis included 57 patients and 26 patients (14 sepsis and 12 trauma patients) were diagnosed DIC by JAAM DIC score. DIC = disseminated intravascular coagulation, JAAM = Japanese Association for Acute Medicine.

There were 96 cases matched with the inclusion criteria in this study (Fig. 1). Of 96, however, 39 patients, including 8 for LOS of less than 2, 5 for out-of-hospital cardiac arrest, 3 for skin burn injury, 1 for electrical injury, and 22 for ISS of less than 16 were excluded. Finally, 57 patients (30 sepsis patients and 27 trauma patients) were assigned to primary analysis.

### 3.1 Demographics and clinical characteristics between sepsis and trauma cases

According to univariate analysis in Table 1, significantly younger age was observed in trauma cases (*P* = 0.001). There was no statistical difference in gender distribution. More patients with circulatory insufficiency tend to be found in sepsis cases and SIRS score was significantly higher in sepsis cases than trauma cases (*P* = 0.001). On the other hand, DIC score, presence of DIC, APACHEII, and SOFA score were not significantly different in the 2 groups. In addition, clinical outcomes did not show statistical significance either.

**Table 1:**
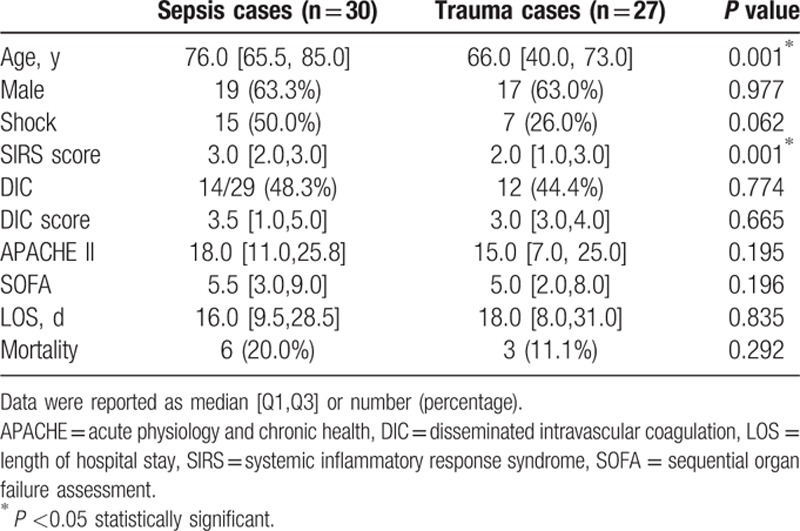
Demographics and clinical outcome in each group.

List of infection site and characteristics of trauma injury are shown in Table 2. More than half of sepsis cases were derived from intraabdominal infection. Trauma patients tend to have multiple injuries. All trauma patients enrolled in this study were caused by blunt injury. Among all patients, 75% of them had severe chest trauma injuries with other injuries and 50% of them had severe head injuries with other injuries. Median ISS was 27.0, RTS 7.55, and Ps 87.4 in this group.

**Table 2:**
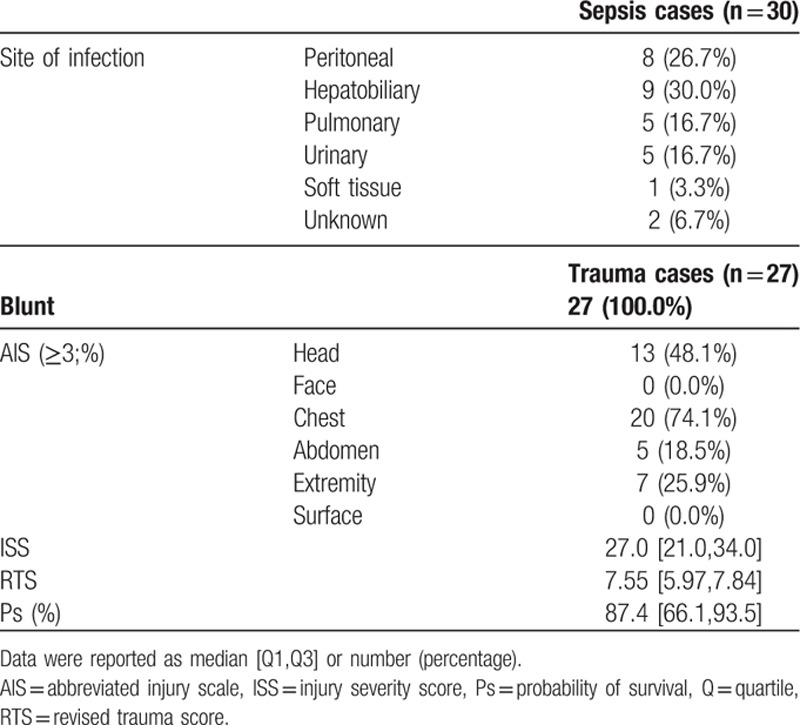
Site of infection and trauma profile on admission in every group.

### 3.2 Differences within septic DIC and traumatic DIC cases

Of 57 patients, 14 sepsis patients and 12 trauma patients were diagnosed with DIC by JAAM DIC score (Fig. 1). Next, we analyzed the differences between 2 DIC groups (Tables 3–5). The same tendency with statistical difference was confirmed about age (*P* = 0.042) (Table 3). The Septic DIC (S-DIC) group showed a significantly higher DIC score than the traumatic DIC (T-DIC) group (*P* = 0.009). No other parameters on gender distribution, rate of shock state, SIRS, APACHEII, SOFA score, or clinical outcomes showed statistical significance between each group.

**Table 3:**
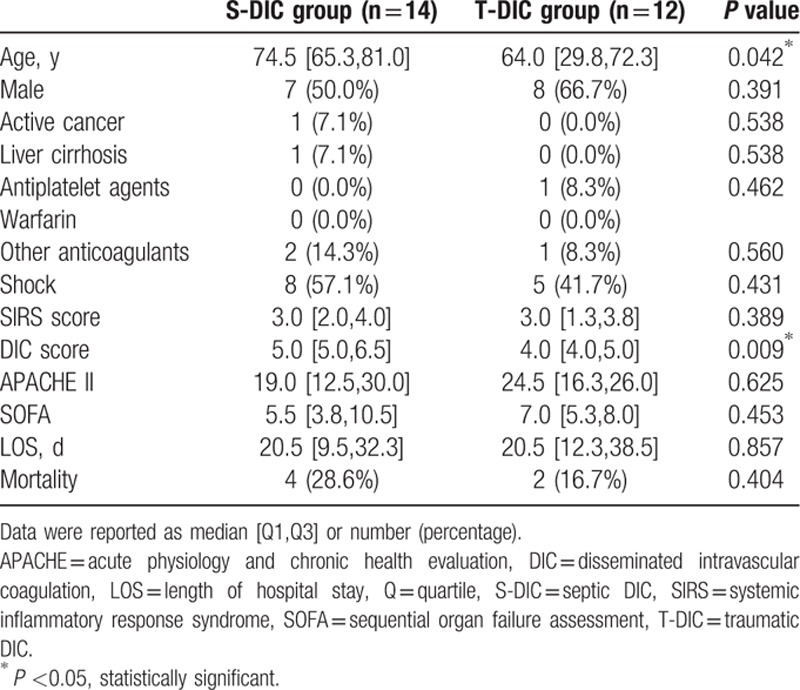
Demographics and clinical outcome in each DIC group.

**Table 4:**
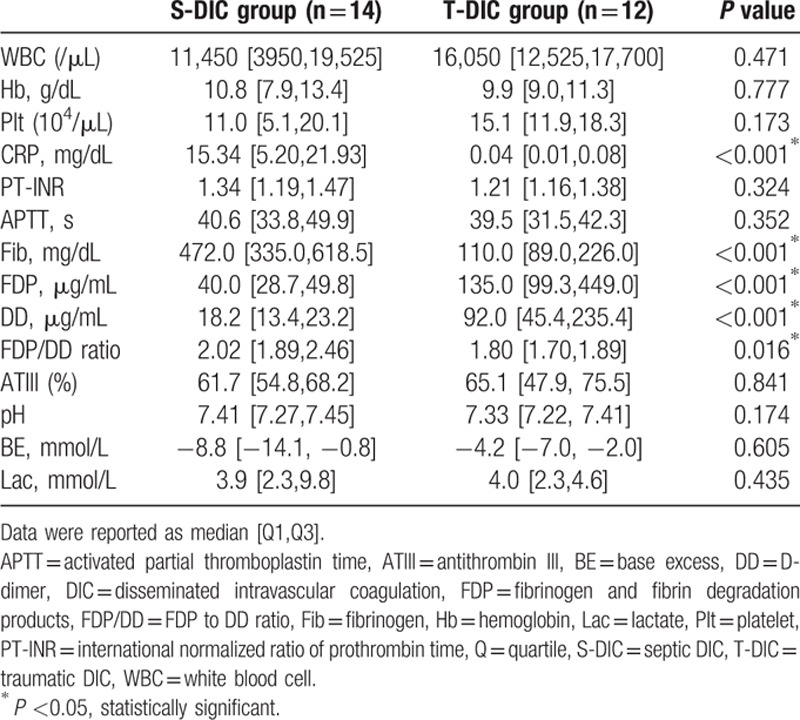
Complete blood count, chemistry, standard coagulation test, and blood gas analysis in each DIC group.

**Table 5:**
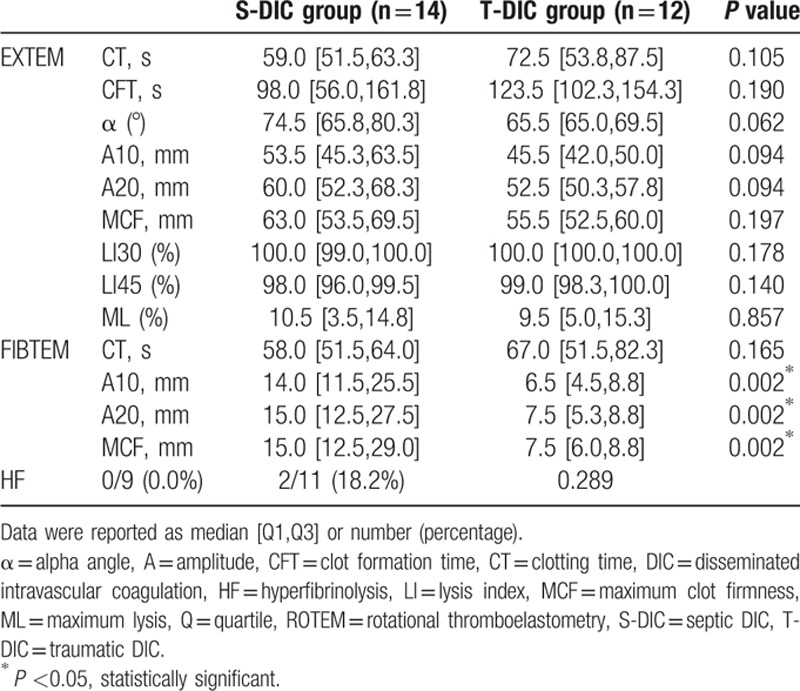
ROTEM (EXTEM/FIBTEM) test in each DIC group.

Hematologic examination revealed that the S-DIC group showed significantly higher CRP (*P* < 0.001), higher Fib (*P* < 0.001), lower FDP (*P* <0.001), lower DD (*P* < 0.001), and higher FDP/DD (*P* = 0.016) ratio than the T-DIC group (Table 4). No other values were statistically significant.

The ROTEM findings in Table 5 showed that A10, A20, and MCF in FIBTEM test were significantly higher in the S-DIC group compared with those in the T-DIC group (*P* = 0.002). In the EXTEM test, higher clot amplitude and higher α angle were confirmed in the S-DIC group, although it was not statistically significant. The LI 30, LI45, and ML, which reflect the degree of fibrinolysis, did not show any statistical differences between the 2 groups.

## 4 Discussion

The present study identified that there were clear statistical differences between sepsis and trauma with similar clinical severities in subjects of CRP, fibrinogen, FDP, DD, and amplitude of blood clot in FIBTEM test. Although significantly higher FDP and DD were observed in septic DIC patients, 2 of the parameters in the JAAM DIC score, it is difficult to distinguish the presence of septic DIC from traumatic DIC patients because these 2 parameters are elevated in both infection and severe injury. It is the same case with CRP value.

On the other hand, this study showed that changes in fibrinogen values between septic DIC and traumatic DIC were completely opposite. An acquired hypofibrinogenemia was observed in trauma patients due to secondary hypercoagulability, acidosis, dilution, massive bleeding, and hypothermia.^[15]^ Indeed, lower fibrinogen level was known as a strong independent risk factor for trauma death.^[16]^ The recent European guideline strongly recommends an early administration of fibrinogen concentrate or cryoprecipitate in the case of plasma fibrinogen level of less than 150 to 200 mg/dL or significant bleeding with functional fibrinogen deficit by thromboelastometry.^[17]^ On the other hand, plasma fibrinogen, known as an acute phase protein, increases in sepsis patients.^[18]^ Recent evidences on the mechanisms of sepsis-related hyperfibrinogenemia have focused on the relationships between inflammation and activation of coagulation.^[19,20]^ Endothelial cells activated by inflammatory cytokines stimulate the extrinsic coagulation pathway. These cells are then able to express adhesion molecules and growth factors, and are directly involved in fibrin formation in sepsis patients.^[19]^ Systemic inflammation-associated coagulopathy is strongly related to organ dysfunctions and clinical outcome in critically ill patients.^[20]^

In the present study, ROTEM analysis revealed that only clot firmness in FIBTEM test was statistically different between the 2 groups. The clot firmness in FIBTEM test is reported to demonstrate a strong correlation with plasma fibrinogen value in cardiovascular surgery, liver transplantation, and trauma surgery.^[21–23]^ Furthermore, the ROTEM using citrated whole blood sample is interpreted to be more pathophysiological compared with the standard coagulation test that is performed with plasma samples.^[24]^ As stated above, we consider that elevation of fibrinogen value is the most prominent feature to distinguish septic and traumatic DIC.

In Japan, the DIC is generally categorized into 3 types by the degree of fibrinolysis compared with existing hypercoagulability: asymptomatic type, marked bleeding type, and organ failure type.^[25]^ Trauma and sepsis have opposite concepts on the fibrinolytic status; however, we could not confirm the statistical differences between them in the degree of fibrinolysis by this thromboelastometrical analysis.^[25–27]^

Higher FDP/DD ratio is related to massive bleeding (cut off: 1.99) and ICU mortality (1.61) in patients with severe trauma, supporting that this dissociation implies the presence of hyperfibrinolysis.^[28]^ However, the present study showed significantly higher FDP/DD ratio in septic DIC compared with traumatic DIC. Although there was a specific reason that can explain these results, this ratio needs to be evaluated on a case-by case basis in consideration of each underlying condition.

There are some potential limitations to this retrospective study, which should be improved, in future studies. A smaller sample size may attribute to a possible selection bias (almost all the samples were performed by 1 doctor and both populations were not statistically equal to each other). Therefore, future prospective investigations involving larger sample size and statistical adjustment will be warranted. Moreover, fibrinolysis dominant DIC (marked bleeding type) are present on admission day in traumatic DIC patients, and this fibrinolytic disorder gradually tends to be suppressed due to persistent plasminogen activator inhibitor 1 (PAI-1) elevation (organ failure type) a few days later.^[29]^ It is important to evaluate the coagulation and fibrinolytic status repeatedly in severely injured patient with high risk of a septic event. In the future, these values will be able to early detect sepsis and abnormalities of the coagulation/fibrinolytic system in complicated patients with trauma or infection.

In conclusion, this study demonstrates hematological differences between septic and traumatic DIC patients. Out of the statistically different parameters, the plasma fibrinogen level and fibrinogen-based clot firmness in whole-blood test revealed obvious statistical difference in this study population.
